# Gamma-interferon exerts a critical early restriction on replication and dissemination of yellow fever virus vaccine strain 17D-204

**DOI:** 10.1038/s41541-017-0039-z

**Published:** 2018-01-23

**Authors:** L. K. Metthew Lam, Alan M. Watson, Kate D. Ryman, William B. Klimstra

**Affiliations:** Center for Vaccine Research, Department of Microbiology and Molecular Genetics, University of Pittsburgh, 3501 Fifth Avenue, Pittsburgh, PA 15261 USA

## Abstract

Live attenuated viruses are historically among the most effective viral vaccines. Development of a safe vaccine requires the virus to be less virulent, a phenotype that is historically arrived by empirical evaluation often leaving the mechanisms of attenuation unknown. The yellow fever virus 17D live attenuated vaccine strain has been developed as a delivery vector for heterologous antigens; however, the mechanisms of attenuation remain elusive. The successful and safe progress of 17D as a vaccine vector and the development of live attenuated vaccines (LAVs) to related flaviviruses requires an understanding of the molecular mechanisms leading to attenuation. Using subcutaneous infection of interferon-deficient mouse models of wild type yellow fever virus (WT YFV) pathogenesis and 17D-mediated immunity, we found that, in the absence of type I IFN (IFN-α/β), type II interferon (IFN-γ) restricted 17D replication, but not that of WT YFV, by 1–2 days post-infection. In this context, IFN-γ responses protected 17D-infected animals from mortality, largely restricted the virus to lymphoid organs, and eliminated viscerotropic disease signs such as steatosis in the liver and inflammatory cell infiltration into the spleen. However, WT YFV caused a disseminated infection, gross liver pathology, and rapid death of the animals. In vitro, IFN-γ treatment of myeloid cells suppressed the replication of 17D significantly more than that of WT YFV, suggesting a direct differential effect on 17D virus replication. Together these data indicate that an important mechanism of 17D attenuation in vivo is increased sensitivity to IFN-γ stimulated responses elicited early after infection.

## Yellow fever: What makes vaccines safe?

The interferon gamma protein may play a key role in preventing yellow fever vaccine 17D from causing virus-like disease in recipients. The highly effective 17D vaccine is a less virulent form of the virus, but can induce severe disease in rare cases. A research group from the University of Pittsburgh, led by William Klimstra, investigated the impact of the vaccine on mice, as the mechanism by which hosts defend against its disease-causing potential is not fully understood. They found that interferon gamma restricted the replication and spread of the attenuated virus (the vaccine) but not its natural form. This study helps to inform efforts to improve the safety of the 17D vaccine, as well as the other vaccines that use it as a template for prophylaxis against other pathogens.

## Introduction

Yellow fever virus (YFV) is the prototypical member of the *Flavivirus* genus of the *Flaviviridae* family, which includes many other important arthropod-borne pathogens such as dengue, Zika, West Nile, and Japanese encephalitis viruses. YFV is estimated to cause 200,000 cases of disease and 30,000 mortalities annually. Historically, YFV has caused devastating outbreaks in North and South America, Africa, and Europe but is currently endemic to sub-Saharan Africa and South America. However, a recent YF outbreak in Angola led to atleast 300 deaths^1^ and 10 symptomatic traveler-associated cases in China,^2^ suggesting that YFV is still a problem in the modern world and threatening to expand geographically.^3^ Mosquito abatement programs and the live-attenuated YFV vaccine strain 17D remain the primary strategies for controlling YF outbreaks.^4^

The YFV vaccine strain 17D is arguably one of the most effective vaccines ever developed. A single dose of 17D results in seroconversion in 95% of vaccinees within a week and can offer essentially life-long immunity against YFV infections.^5,6^ While the safety record of 17D is exemplary, severe adverse events do occur with low frequency, and these can involve both viscerotropic and neurotropic manifestations of virus disease. Regardless, due to the overall safety and effectiveness of 17D, multiple candidate vaccine vectors have been created using its genetic backbone and are currently being tested as a delivery system for antigens of other flaviviruses or other pathogens.^7,8^

Despite being safe and effective, the mechanisms for attenuation and immunogenicity of 17D remain elusive. The 17D vaccine strain was derived by Max Theiler in 1930s through serial passaging wild-type (WT) YFV strain Asibi in tissue culture, resulting in loss of viscerotropism and neurotropism in non-human primates.^9^ Multiple substrains of 17D, including 17D-204, 17D-213, and 17DD have been generated and used for vaccination,^10^ with similar efficacy.^11^ An average of 48 nucleotide and 20 amino acid changes distinguish 17D from its parent Asibi strain.^12,13^ The attenuation of 17D is attributed to genetic differences in both structural and non-structural proteins, but the role of specific mutations in the attenuation phenotype are not well characterized. Lee et al. suggested that a positive-charge mutation in the 17D envelope (E) protein that leads to enhanced glycosaminoglycan binding, contributes to attenuation, but attenuation likely involves multiple 17D loci.^14^ It was recently demonstrated that E protein mutations allow 17D to enter susceptible cells through clathrin-independent pathways, leading to enhanced antiviral response in vitro.^15^ We recently reported that both neutralizing antibody and CD4^+^ T cells are important in 17D-mediated protection from lethal WT YFV infection in a viscerotropic disease model.^16^ However, questions remain regarding the specific virus-host interactions that are modified by the 17D attenuating mutations and the mechanisms through which 17D induces highly protective immune responses. Understanding of the mechanisms of 17D attenuation will promote informed design of live attenuated versions of other flaviviruses and vaccines derived from other virus types.

To assess the specific host interactions affected by 17D attenuating mutations, we have used a pathophysiologically relevant mouse model to study host-pathogen interactions of YFV. Many WT flaviviruses, including YFV, replicate extremely poorly in mice with a functioning type I IFN system, precluding study of the roles of other host factors in their pathogenesis. Flaviviruses are more effective at antagonism of human type I IFN signaling than the analogous mouse responses.^17,18^ Indeed, mice deficient in the type I interferon (IFN-α/β) receptor (AB6) are susceptible to viscerotropic disease and lethality after subcutaneous (s.c.) infection with wild-type YFV strains Asibi and Angola71.^19^ Interestingly, s.c. infection of 17D in AB6 mice, which mimics vaccination, does not cause discernable disease and results in life-long immunity against challenge of WT virus strain Angola71.^16^ However, mice lacking both type I and type II IFN receptors (AGB6) are susceptible to lethal infection by 17D by either subcutaneous or intra-peritoneal routes.^14,19,20^ The fact that the additional deficiency of type II IFN (IFN-γ) receptors renders 17D infection lethal in AB6 mice suggests that the type II IFN system plays a critical role in attenuation of 17D in vivo.

To begin to understand the effects of IFN-γ on 17D, we have compared infection of AB6 and AGB6 mice. IFN-γ restricted virus replication and dissemination early during infection and enhanced 17D-204 virus clearance late after infection. Antiviral gene induction and cytokine production was also influenced by the presence or absence of type II IFN signaling. Importantly, we found that 17D-204 was more sensitive than WT virus strain Angola71 to the antiviral state induced by IFN-γ in vitro in specific myeloid cell subtypes likely important to YFV pathogenesis. This is suggestive that IFN-γ sensitivity is a primary attenuation mechanism for 17D in vivo. Finally, 17D-204 infection resulted in liver stress and histological abnormalities in the brains of AGB6 but not AB6 mice, indicating that both viscerotropic and neurotropic diseases occurred in the absence of IFN-γ signaling and revealing a possible application for the AB6 model in understanding vaccine-associated viscerotropic and neurotropic adverse events.

## Results

### IFN-γ attenuates 17D-204 but not virulent YFV in vivo

**Fig. 1:**
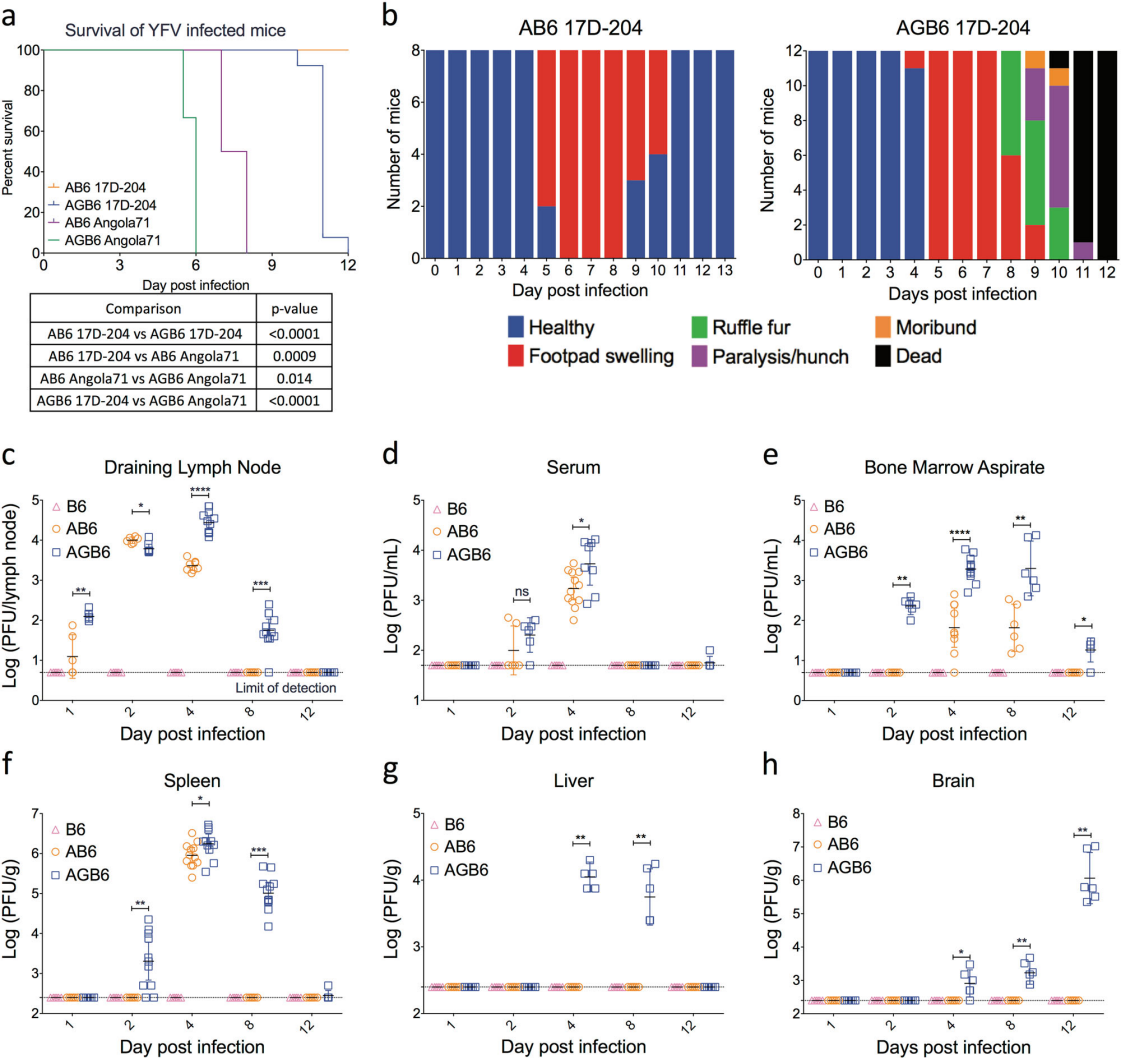
IFN-γ attenuates 17D-204 in vivo. Survival (**a**) and symptoms (**b**) of diseases of YFV-infected mice were monitored daily. **c**–**h** 17D-204 replication kinetics in vivo. **c** Popliteal draining lymph node, **d** serum, **e** 1 mL bone marrow aspirate, **f** spleen, **g** liver, **h** brain. Data in (**a**) are analyzed with log-rank test. Data in **c**–**h** are presented in geometric mean ± 95% CI, 1 out of 3 independent experiments is shown. (*p < 0.05; **p < 0.01; ***p < 0.005; ****p < 0.001; multiple *t*-test, corrected by Holm–Sidak method. 17D-204, *n* ≥ 6, Angola71, *n* = 4)

To explore the role of IFN-γ, we compared pathogenesis of 17D-204 in AB6 and AGB6 mice. Groups of 6-week-old AB6 and AGB6 mice were infected subcutaneously with 10^4^ PFU of YFV 17D-204 or Angola71 in both of the hind limb footpads. The virulent WT strain Angola71 was used as control. As reported previously,^16,19^ Angola71-infected mice experienced severe weight loss and disease, requiring euthanasia (AST 7.5 +/− 0.6 dpi), whereas 17D-204 was uniformly non-lethal in AB6, and 17D-204-infected AGB6 mice experienced severe weight loss and eventually succumbed to disease (AST 10.75 +/− 0.5 dpi) (Fig. 1a). Both 17D-204-infected AB6 and AGB6 had footpad swelling. In addition, 17D-204-infected AGB6 displayed signs of brain infection and neurologic disease including hind-limb paralysis at the late stage of infection before euthanasia (Fig. 1b).

### IFN-γ controls virus replication in vivo and protects mice from viscerotropic and neurotropic diseases

Because IFN-γ influences survival of mice after 17D-204 infection, we hypothesized that virus replication is reduced and restricted in the presence of IFN-γ. Thus, we compared 17D-204 virus replication kinetics in different tissues in the AB6 and AGB6 mice. In parallel, tissues from 17D-204-infected wild-type (C57BL/6) mice were harvested but no infectious virus was recovered from any tissue sampled (Fig. 1c–h).

The regional lymph node draining the site of infection (DLN) is one of the earliest sites for flavivirus replication and important for seeding viremia and dissemination.^19^ At 1 dpi, 17D-204 virus was recovered from the DLN in both AB6 and AGB6 mice. In the absence of the IFN-γ response, 17D-204 virions were more abundant as early as at 1 dpi (Fig. 1c). Serum viremia could be observed by 2 dpi; however, no significant difference between serum viremia was observed in AB6 versus AGB6 at 2 dpi (Fig. 1d). 17D-204 virions were detectable in bone marrow aspirate and spleen at 2 dpi only in the absence of IFN-γ response, but by 4 dpi, high virus titers were observed in the spleen, bone aspirate, non-draining LN, adrenal glands, kidneys, and heart in both types of mice (Fig. 1e, f and S1), although, higher virus titers were observed in AGB6 mice in most of these tissues. Interestingly, infectious virus particles were recovered from liver and brain in AGB6 mice but never with AB6 mice (Fig. 1g-h). In the presence of IFN-γ responses, virus clearance was observed in most organs by 8 dpi, but virions were still detected in all sampled organs in AGB6 mice at this time. Despite lacking both type I and type II IFN, virus clearance was observed in most of the non-CNS tissues in AGB6 mice by 12 dpi, before they succumbed to disease. In contrast, virus replication in brain increased through 12 dpi (up to 10^6^ PFU/g) in the AGB6 mice (Fig. 1h), corresponding to the time when neurological signs were first observed (Fig. 1b). Overall, in the presence of IFN-γ, 17D-204 replication was controlled as early as 1 dpi. Some dissemination of virus still occurred in AB6 mice, but the peak viral load was significantly lower than that of AGB6 mice. Virus tropism was also restricted by IFN-γ, most notably from liver and brain, and virus clearance was observed in most tissues by 8 dpi in AB6 mice. In the absence of IFN-γ responses, virus clearance occurred later and only in non-CNS tissues.

**Fig. 2:**
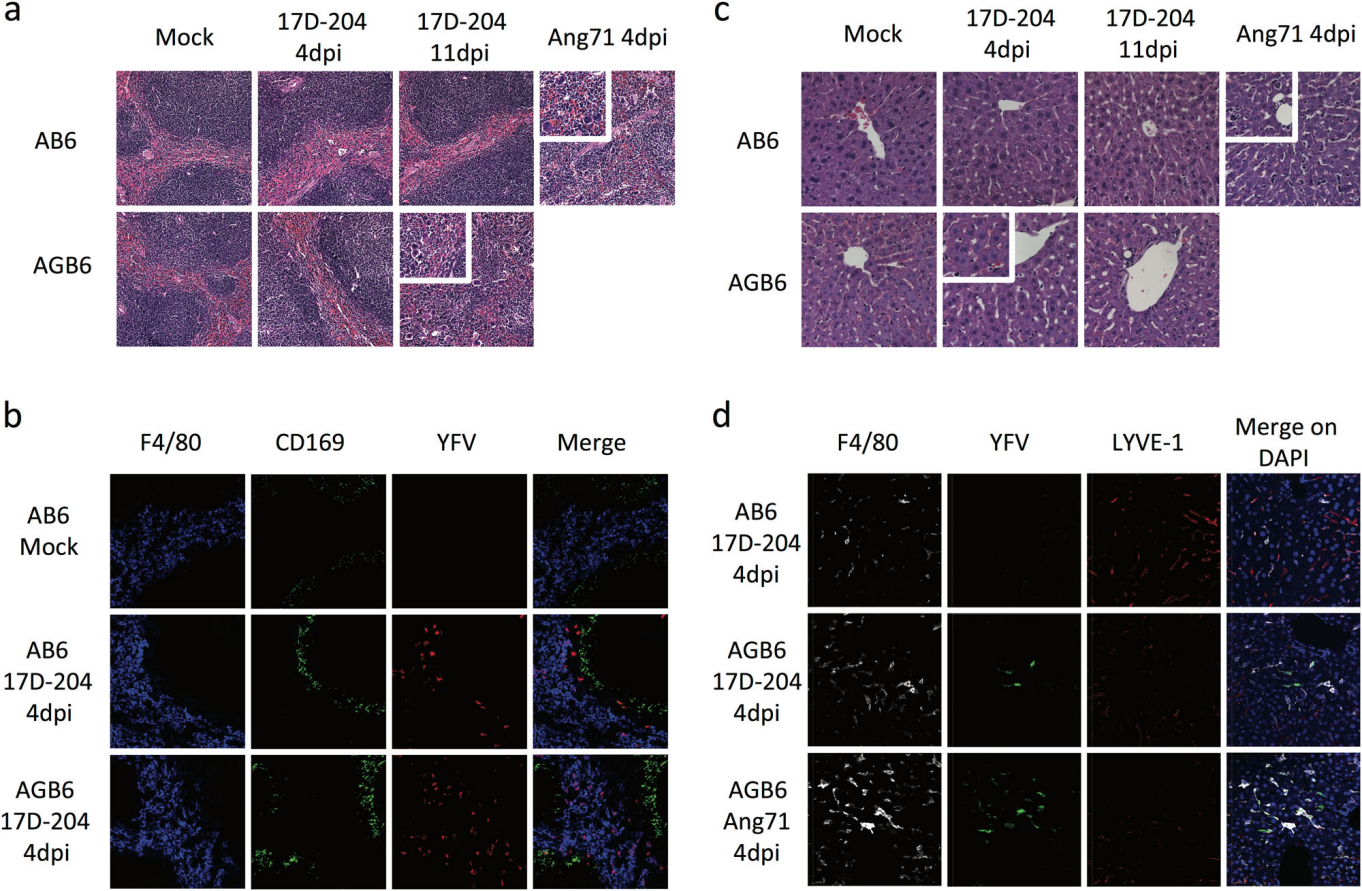
17D-204 causes viscerotropic abnormalities in the absence of IFN-γ. H&E sections (**a**–**c**) antibody-stained frozen sections (**b**, **d**) of spleen (**a**, **b**) and liver (**c**, **d**) were presented. **a** Spleen sections from 17D-204-infected animals were indistinguishable at 4 dpi, whereas Angola71-infected animals display loss of white pulp and red pulp architecture and increase in infiltrating macrophages and neutrophils (inset) at 4 dpi. At 11 dpi, 17D-204 infected AGB6 has increased immune infiltration and extramedullary hematopoiesis (inset) but not AB6 mice. **b** YFV antigen can be detected in the spleen at 4 dpi in both AB6 and AGB6 mice in the red pulp and outer marginal zone area. **c** 17D-204-infected AB6 mice do not display major histological changes but infected AGB6 mice and Angola71-infected animals had microsteatosis (inset) at 4 dpi. In AGB6 mice, microsteatosis only occurs transiently at 4 dpi and resolved by 11 dpi. Antibody-stained frozen liver sections (**d**) revealed that YFV antigen could only be detected in infected AGB6 mice at 4 dpi but not AB6 mice, parallel to titer data in Fig. 1g. Original magnification = 40x, *n* = 4

Because 17D is known to cause viscerotropic and neurotropic infection in vaccine-associated severe adverse events (SAE) cases,^21,22^ we sought to determine if tissue pathology could be detected after 17D infection in the absence of an IFN-γ response. We investigated tissue pathology at 4 dpi, which is the peak of viral replication in most visceral organs in both AB6 and AGB6 mice and 11 dpi, when the AGB6 animals displayed neurologic signs before succumbing to disease. At 4 dpi, hematoxylin and eosin (H&E) stained spleen sections (Fig. 2a) of 17D-204-infected AB6 and AGB6 mice were indistinguishable from mock-infected animals. In contrast, spleens of Angola71-infected mice displayed loss of splenic architecture and increase in inflammatory infiltrates, similar to our previous report.^19^ Despite the lack of titerable virus in the spleen at 12 dpi (Fig. 1f), spleens of 17D-204-infected AGB6 had increased numbers of inflammatory infiltrates and exhibited a loss of splenic architecture (Fig. 2a inset). Immunostaining of spleen sections from infected AB6 and AGB6 mice demonstrated the presence of YFV antigens in F4/80^+^ cells and cells in the outer marginal zone (Fig. 2b) at 4 dpi at a similar level. H&E-stained liver sections revealed microsteatosis occurring only in the liver of 17D-204-infected AGB6 mice at 4 dpi (Fig. 2c) a time at which virions were detectable in the liver (Fig. 1g), and thus, indicative of a viscerotropic phase of 17D-204 infection in the absence of IFN-γ responses. Moreover, YFV antigen could be detected in some F4/80^+^ cells in the liver of infected AGB6 mice, suggesting that Kupffer cells were infected in these animals (Fig. 2d). Corresponding to the time of neurological signs in 17D-204-infected AGB6 mice (11 dpi, Fig. 1b), we observed an increase in immune infiltration to the cerebral cortex (Fig. S2a) and virus infected cells in the cerebral cortex and cerebellum of the brain (Fig. S2b-c), indicative of neurotropic disease. No such phenomena were detectable in 17D-204-infected AB6 animals. Overall, our histology data demonstrates that IFN-γ restricts 17D-204 dissemination and protects mice from viscerotropic and neurotropic diseases.

### Cytokine induction by 17D-204 is reduced in the absence of IFN-γ signaling

**Fig. 3:**
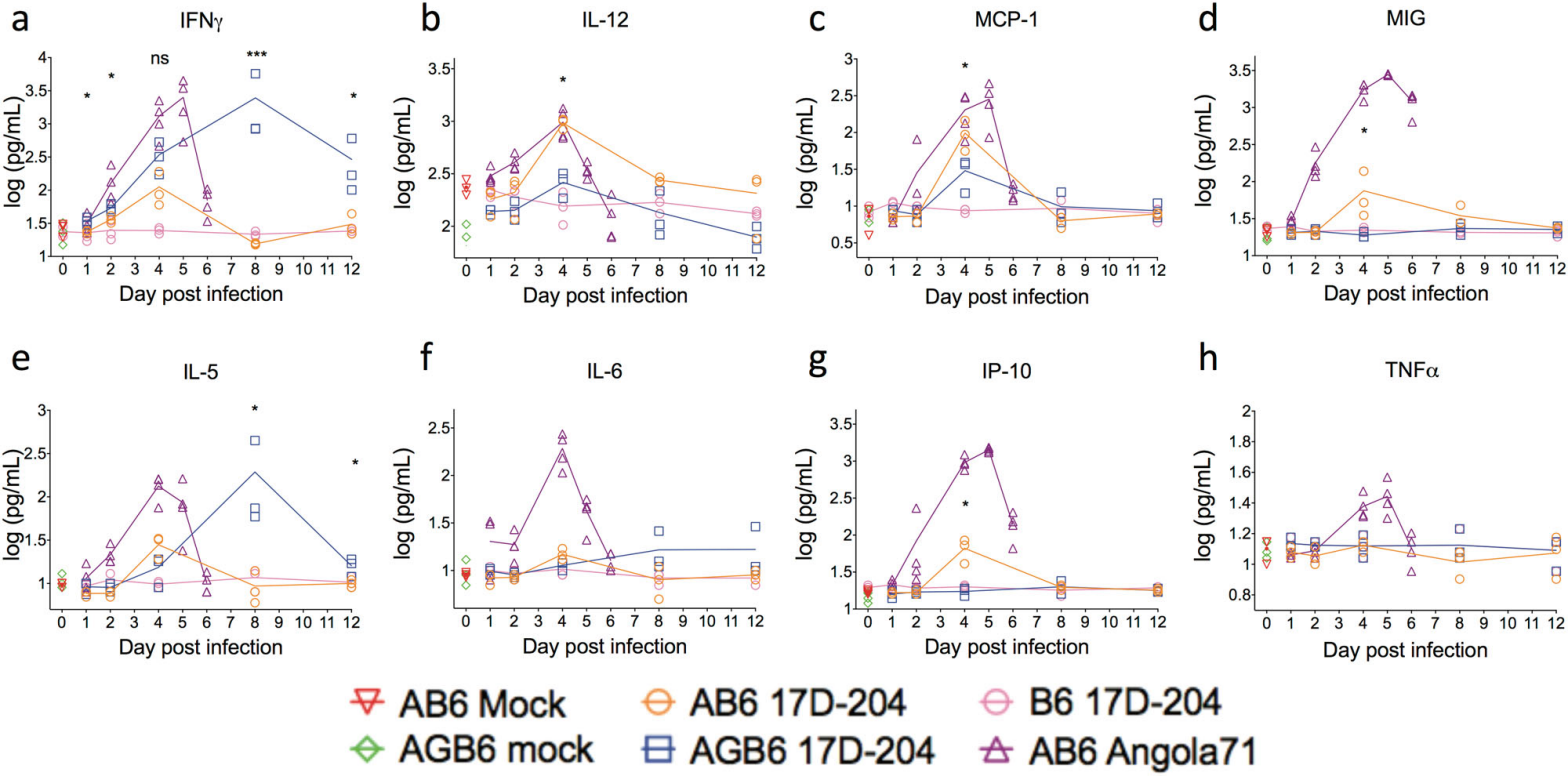
Cytokine response of 17D-204-infected animal. Serum cytokines IFN-γ (**a**), IL-12 (**b**), MCP-1 (**c**), MIG (**d**), IL-5 (**e**), IL-6 (**f**), IP-10 (**g**), and TNFα (**h**) were quantified using Cytokine 20-Plex Mouse Panel bead-based assay. *n* = 3 (17D-204) or 5 (Angola71). Data are presented in geometric mean ± 95% CI. Statistical comparisons are determined between 17D-204-infected AB6 and AGB6 mice. (*p < 0.05, **p < 0.01, ***p < 0.005; multiple *t*-test, corrected by Holm–Sidak method)

IFN-γ is central to activation of T cells and macrophages, and induces production of proinflammatory cytokines.^23^ Therefore, we hypothesized that the absence of IFN-γ would impact cytokine production during 17D infection. To this end, serum cytokine levels in infected mice were measured at various times post-infection using the Cytokine 20-plex Mouse Panel (Fig. 3). In AB6 mice infected with 17D-204, serum levels of IFN-γ, IL-12p40, MIG, MCP-1, and IP-10 were elevated transiently versus uninfected controls on day 4 pi, corresponding to the peak spleen virus titer (Fig. 1f). While AGB6 mice also exhibited a slight increase in serum IL-12 and MCP-1 levels on day 4pi, serum IFN-γ levels were highly elevated in comparison with control or AB6 mice on this day and at days 8 and 12pi. Wild-type YFV-infected AB6 mice produced higher levels of serum cytokines (IFNγ, MIG, MCP-1, IL-4, IL-5, IP-10, and TNFα) more rapidly than in 17D-infected AB6 or AGB6 mice (Fig. 3), possibly associated with the severe viscerotropic disease and rapid death of the WT virus-infected animals.^19^ The cytokines that are more significantly upregulated in 17D-204-infected AB6 but not AGB6 mice, i.e. IL-12, MCP-1, MIG, and IP-10, are all IFN-γ-inducible, suggestive of a central role for IFN-γ in protecting AB6 mice from 17D-204 infection through cytokine induction. Similar to our observations, in adult human male vaccinees receiving YFV vaccine sub-strain 17DD, serum levels for MCP-1, MIG, IP-10, and IFN-γ were reported to be elevated.^24^

### Antiviral gene induction is limited in 17D-204-infected AGB6 mice

**Fig. 4:**
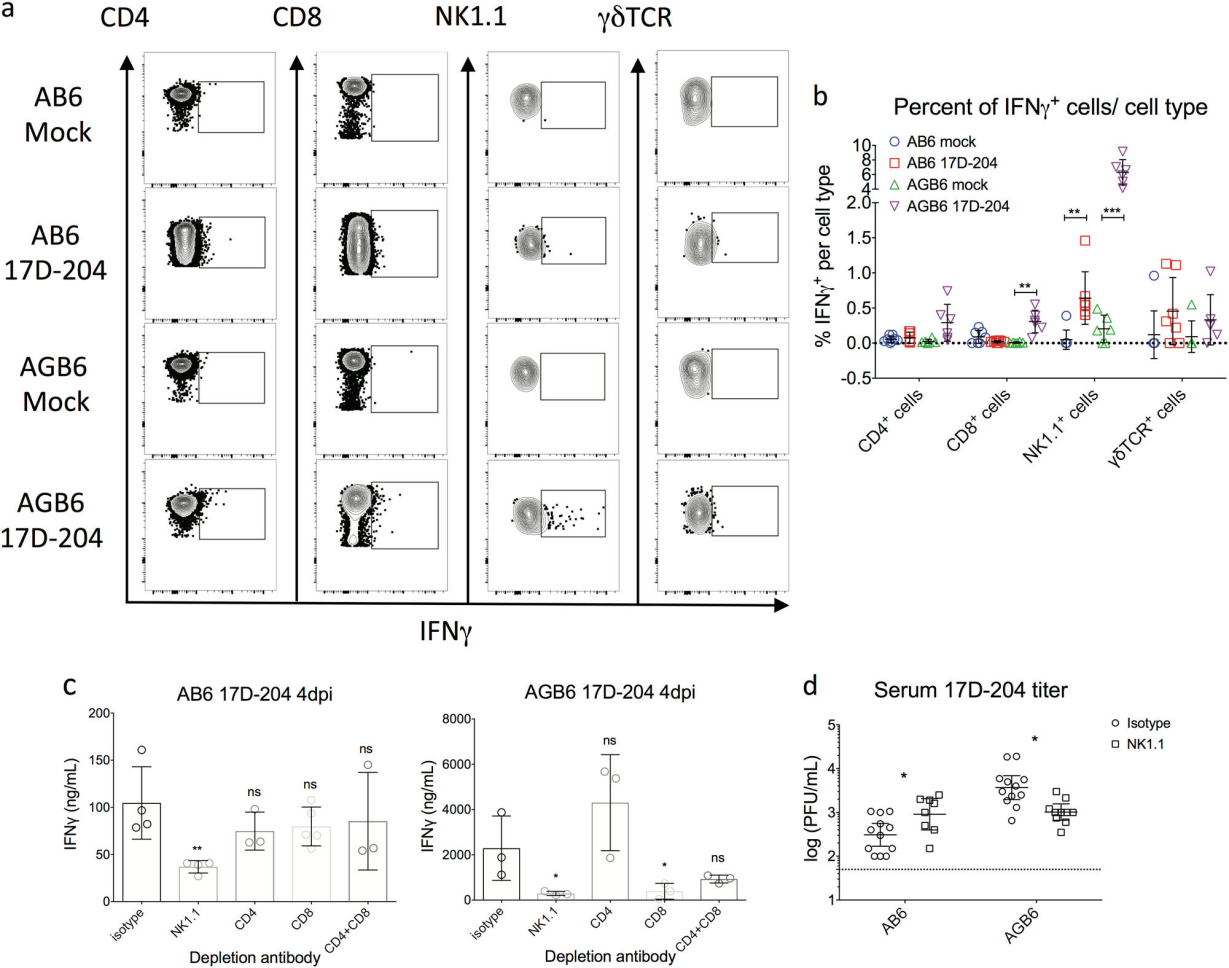
NK cells are important for IFN-γ production. **a**, **b** IFN-γ production in draining lymph nodes cells at 3 dpi. Single cell suspensions were prepared for flow cytometry analysis to identify cell types producing IFN-γ. (*n* = 6, 1 out of 3 independent experiments is shown; see Fig. S4 for gating strategy.) **c**, **d** Depletion of NK cell led to lower serum IFN-γ levels and enhanced viral replication. **c** ELISA quantification of serum IFN-γ at 4 dpi. **d** Serum virus titer at 4 dpi. Data are presented in mean ± SD in **b**, **c** and geometric mean ± 95% CI in **d** (*p < 0.05, **p < 0.01, ***p < 0.005, ****p < 0.001; multiple *t*-test, corrected by Holm–Sidak method)

IFN-γ has been shown to upregulate and activate cellular and humoral immunity,^25,26^ but it can also be directly antiviral versus YFV in many cell types through upregulation of genes that encode antiviral effector proteins such as IFN-stimulated genes (ISGs).^27^ Since IFN-γ inhibition of 17D-204 occurs at early times after infection of AGB6 mice, we attempted to determine if differences in tissue-specific expression of IFN-γ or ISGs could be detected in the absence of IFN-γ. Because we observed inhibition of 17D-204 replication and dissemination before 4 dpi, we examined IFN-γ transcript induction from 1 dpi to 4 dpi. Robust upregulation of the IFN-γ gene was observed as early as 2 dpi in lymph nodes but not spleens of both AB6 and AGB6 mice (Fig. S3a). Using flow cytometry (Fig. 4a, b), we observed induction of IFN-γ in NK1.1^+^ cells in the DLN, similar to a previous report.^28^ Despite the lack of IFN-γ induction in the spleen (Fig. S3a), various antiviral genes including IGTP, IFIT1, and IFIT2 were upregulated in the spleens of 17D-204-infected AB6 mice more robustly than that of AGB6 mice, indicative of IFN-γ-dependent gene induction and likely a systemic effect of IFN-γ production (Fig. S3b).

To confirm the role of NK cells in IFN-γ production in vivo, we performed antibody-mediated depletion of NK1.1^+^, CD4^+^-, or CD8^+^-T cells in 17D-204-infected mice. In both AB6 and AGB6 mice, NK1.1^+^ cell depletion led to a reduction in serum IFN-γ levels at 4 dpi (Fig. 4c), supporting the flow cytometry data identifying NK1.1^+^ cells as a primary source of IFN-γ. Depletion of CD8^+^ T cells in AGB6, but not AB6, animals also led to reduction in serum IFN-γ levels. NK1.1^+^ cell depletion also resulted in enhanced 17D-204 replication in AB6 mice (Fig. 4d), likely due to less IFN-γ production. However, depletion of NK1.1^+^ cells resulted in reduced virus titer in AGB6 mice. The latter may be due to the recently documented replication of 17D in NK1.1^+^ cells in the absence of IFN-α/β and IFN-γ signaling.^29^

### 17D-204 is more sensitive to an IFNγ-induced antiviral state than WT-YFV

**Fig. 5:**
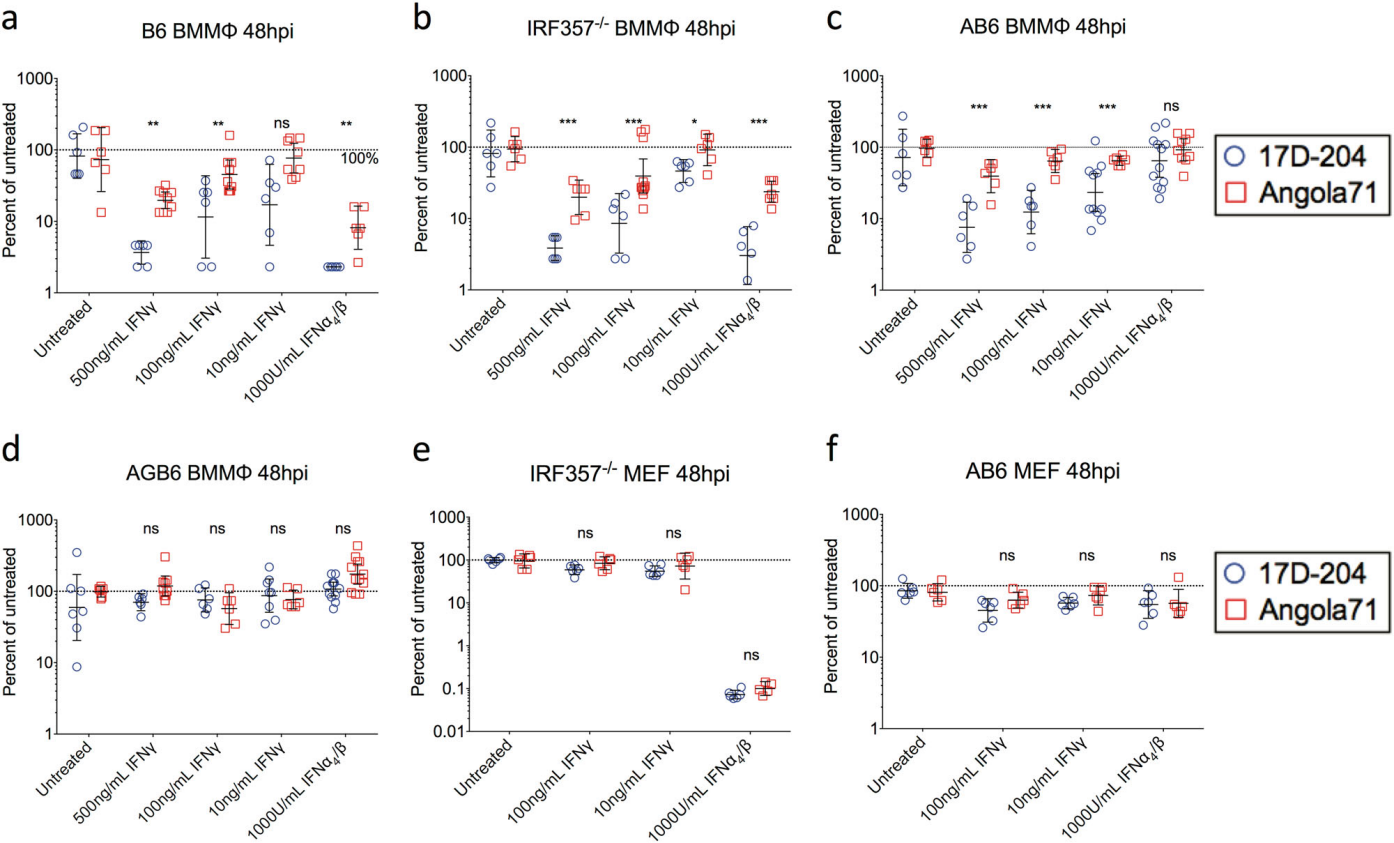
17D-204 is more sensitive to IFN-γ-induced antiviral states than wild-type strain Angola71 in myeloid cells. Indicated cell types were treated with designated IFN 12 h prior to infection with 17D-204 or Angola71 at MOI = 0.1. (*n* = 6, 1 out of 3 independent experiments is shown.) At 48 hpi, supernatants were harvested and infectious virus particles were quantified by focus forming assay on Vero cells. **a**–**d** Bone marrow-derived macrophages and **e**, **f** mouse embryonic fibroblasts from various strains of mice. Foci titers (FFU/mL) were normalized to that of untreated cells, and data are presented in mean percentage ± SD. Corresponding foci titers are available in Fig. S5. (*p < 0.05, **p < 0.01, ***p < 0.005, ****p < 0.001; multiple *t*-test, corrected by Holm–Sidak method)

Based on our observations that (1) AGB6 mice succumbed to 17D-204 infection but not AB6 mice (Fig. 1a); (2) 17D-204 replicated to higher titer and disseminated faster in AGB6 mice than AB6 (Fig. 1c–h); (3) IFN-γ induction in infected AB6 and AGB6 mice occurred as early as 2 dpi (Fig. S3); (4) Angola71-infected AB6 mice produced higher levels of serum IFN-γ than 17D-infected AB6 (Fig. 3), yet virus infection was not controlled in the Angola71-infected mice; (5) Angola71-related mortality was delayed in AB6 versus AGB6 mice (Fig. 1a); and (6) reports that IFN-γ inhibited flavivirus infection in vitro,^30,31^ we hypothesized that an IFN-γ-induced antiviral state can inhibit YFV replication in vitro and in vivo and that enhanced sensitivity to the IFN-γ antiviral state is a prominent attenuation mechanism of 17D. To test this hypothesis, we compared virus replication of 17D-204 and Angola71 in various relevant cell types treated with IFN-γ or IFN-α/β. Consistent with the hypothesis, we observed a dose-dependent inhibition of 17D-204 and Angola71 virus replication by IFN-γ in bone marrow-derived macrophages (BMMΦ) and dendritic cells (BMDC) derived from C57BL/6 mice (Fig. 5 and S5-6). In addition, 17D-204 was significantly more inhibited than Angola71 by an equivalent dose of IFN-γ or IFN-α/β. Since basal replication, which may be different between the viruses, can potentially induce different levels of IFN-α/β in cultured cells and alter sensitivity measurements, we performed the same experiments using cells that lack the ability to produce (IRF3x5x7^−/−^ cells) or respond to (AB6 cells) IFN-α/β (Fig. 5b-c). Cells derived from AGB6 mice, which cannot respond to either type of IFN, were also included as negative controls (Fig. 5d). While 17D-204 and Angola71 multiply to higher titers in IRF3x5x7^−/−^ and AB6 BMMΦ than BMMΦ derived from C57BL/6 mice, 17D-204 is still significantly more inhibited by IFN-γ than Angola71. Interestingly, we did not observe significant differences between the viruses to either IFN-α/β or IFN-γ treatment in immortalized mouse embryonic fibroblasts (MEFs) (Fig. 5e-f), suggesting that 17D-204 and Angola71 are differentially inhibited by IFNγ-induced antiviral state in a cell type-specific manner.

## Discussion

Despite the success and widespread use of the 17D YFV vaccine strains, the molecular mechanisms for attenuation of 17D remain unclear. One major restriction on understanding YF pathogenesis has been the lack of pathophysiologically relevant and cost-effective small animal models for studying YFV pathogenesis and attenuation. Previously, we reported that mice lacking type I interferon receptor (IFNAR^-/-^) are susceptible to subcutaneous (s.c.) infection of wild-type YFV and display signs of viscerotropic disease similar to human YF, whereas 17D is avirulent in this model system.^19^ In addition, 17D-infection leads to protective immunity against wild-type YFV challenge.^16^ However, we and others have reported that 17D is virulent in mice lacking both type I and type II IFN receptors (IFNAGR^-/-^), suggesting that type II IFN is a critical attenuation factor for 17D in vivo. In this study, we investigated the role of type II IFN in controlling replication of 17D in vitro and in vivo. Our results suggest that type II IFN restricts 17D-204 replication and spread in vivo. Furthermore, 17D-204 is more sensitive to the antiviral activities of IFN-γ than wild-type YFV in myeloid, but not fibroblastic, cells in vitro.

By comparing 17D-204 pathogenesis in AB6 and AGB6 mice, we found 17D-204 replication was largely restricted to lymphoid compartments by type II IFN. In the absence of IFN-γ response, 17D-204 infection in AGB6 mice rapidly disseminated to tissues beyond DLN, and bone marrow. In addition to overall higher viral titers, we observed signs of liver stress and recovered infectious virus particles in the liver on 4 dpi, indicative for a viscerotropic phase of 17D-204 infection with the additional absence of IFN-γ responses. Despite viral clearance in the peripheral tissues, virus accumulated to high titer in the brain associated with signs of neurological disease including immune cell recruitment to the brain and paralysis. Taking together our data and those of other reports,^19,20^ we propose that 17D-infected AGB6 mice could be used to model neurotropic SAEs associated with 17D vaccination. Despite the presence of a brief viscerotropic phase of 17D-204 infection in AGB6 mice, we did not observe cytokine storm comparable to Angola71-infected AB6 mice or extensive damage to the visceral organs (which can be found in viscerotropic SAE patients^32^), except the spleen late during infection. These data indicate involvement of additional or alternative factors, such as host genetics^33,34^ and/or 17D genetic variants,^35^ both of which have been proposed as sources of SAE.

In this report, we examined the role of type II IFN in attenuation of 17D in vivo. Consistent with other studies of 17D vaccination in mice^28^ and non-human primates (NHP)^36^, we observed an early induction of IFN-γ after 17D-204 vaccination in our mouse model. Such early induction of IFN-γ resulted in contemporaneous restriction of 17D-204 in AB6 mice. In both AB6 and AGB6 mice, NK1.1^+^ cells were critical for IFN-γ production; however in AGB6, CD8^+^ T cells (CTL) were also involved. Lymphocyte activation is driven by antigen abundance and the high virus titers in AGB6 mice may have contributed to a more robust CTL activation and IFN-γ production than in AB6 mice. In addition, a recent report has shown that CTL and NK cells can be infected by 17D in the absence of STAT1. In the report^29^, deletion of STAT1 in the hematopoietic compartment rendered mice susceptible to intravenous 17D infection, and enhanced 17D replication in leukocytes from both myeloid and lymphoid lineages was detected in the spleen and circulation. Thus, we speculate that infection of, both CTL and NK cells leads directly or indirectly to IFN-γ secretion. Whereas in AB6 mice, the presence of IFN-γ response partially protects T cells directly through inducing antiviral state and/or indirectly through control of virus titer, leaving NK cells as the major IFN-γ producer.

While the role IFN-γ plays in inducing protective immunity against 17D requires further study, our data suggest one mechanism by which IFN-γ controls 17D is through inducing a direct antiviral response, especially in macrophages and DCs. Cell type-specific effects of IFN-γ have been previously observed. In mice, IFN-γ-induced antiviral state inhibits murine cytomegalovirus more robustly in macrophages than in MEF.^37^ Murine DCs and macrophages also respond to IFN-γ differently.^38^ Furthermore, the maturation state of macrophages affects the binding of STAT1 to IFN-γ-activated promoter sites (GAS).^39^ Clearly, further work is needed to elucidate the role of specific IFN-γ induced antiviral effectors in suppression of 17D replication, in particular, the specific mutations in 17D responsible for the increased IFN-γ sensitivity need to be identified and how these mutations increase susceptibility to individual antiviral effectors. Other than mutations in E protein that confer increased heparan sulfate binding,^14^ the effects of mutations in 17D have yet to be characterized. The mutations M-L36F and NS4B-I95M that differentiate between WT strains and attenuated strains (17D substrains and French neurotropic vaccine strain) are possible candidates for influencing IFN-γ sensitivity.^40^ For M-L36F, substitution of the analogous amino acid in Japanese encephalitis virus results in a mutant deficient in virion maturation and virus particle production.^41^ NS4B from various flaviviruses is an antagonist of IFN signaling and inhibits STAT1 translocation.^42^ It is possible that these mutations, alone or in combination with other 17D mutations, could influence sensitivity to IFN-γ. However, this remains to be investigated. Although IFN-γ exerts an early effect on viral replication and dissemination, peripheral clearance is also observed in AGB6 mice by 12 dpi, which is suggestive of a type II IFN-independent viral clearance mechanism. The nature of this response also needs to be determined as it may be important in minimizing pathological consequences of 17D immunization such as SAEs. Despite the lack of both type I and type II IFN responses, some antiviral genes were upregulated in our model, especially in the DLN and spleen. These genes may be induced directly without type I or type II IFN through RIG-I and MDA5,^43^ toll-like receptors,^44^ or type III IFN,^45^ which may aid eventual viral clearance from the periphery even in AGB6 mice. However, it was recently reported that mice lacking both type I and type III IFN receptors had higher 17D titers in the brain but not other tissues when compared to mice lacking IFNAR alone, suggestive of a neuroprotective role for type III IFN.^46^

One potential criticism of a mouse model lacking type I IFN response is the lack of viral control and impaired induction of adaptive immunity. However, evidence suggests infection of these mice recapitulates important aspects of YFV infection and vaccination, namely, viscerotropic disease during WT virus infection and long-term protective immunity after 17D vaccination.^16,19^ Indeed, robust, protective B and T cell responses to 17D are present in these animals.^16^ In human vaccinees, the presence of type I IFN does not prevent 17D replication and establishment of serum viremia or mild reactions at the site of vaccination. Whereas in C57BL/6 mice, the presence of type I IFN suppresses 17D replication to the point that replicating viruses cannot be detected by conventional assays, and infected mice do not have local reactions such as footpad swelling after infection. This suggests that YFV is more resistant to, or more capable of suppressing, the human type I IFN system than the murine counterpart. In fact, dengue and Zika virus are more effective in antagonizing human innate immune signaling molecules than murine homologs.^17,18,47^ Together, these observations suggest that the type I IFN receptor-deficient mouse is a pathophysiologically relevant model that is valuable in exploring the immunogenicity, pathogenicity, and attenuation mechanisms of different YFV strains. However, type I IFN likely also has an important role in controlling YFV in humans.^48,49^

These studies have revealed that sensitivity of 17D to murine IFN-γ-mediated response protects IFNAR^-/-^ mice from disease and promotes the eventual development of robust and long-lived protective immunity against wild-type YFV challenge^16^ similar to human vaccination. In contrast, the lack of an IFN-γ response renders 17D virulent and leads to viscerotropic and neurologic disease and eventual death, potentially similar to human SAEs. Enhanced sensitivity to IFN or particular ISGs has been documented in several arbovirus LAVs.^50–52^ Thus, the deliberate creation of an IFN-γ sensitive phenotype may be a productive approach to rational design of new LAV candidates against arboviruses.

## Materials and methods

### Ethics statement

Animals were maintained and procedures were performed in accordance with the recommendations in the Guide for the Care and Use of Laboratory Animals of the National Research Council. Protocols 1103456 and 14033545 were approved by the University of Pittsburgh’s IACUC committee. Approved euthanasia criteria were based on weight loss and morbidity.

### Cells lines

Vero (ATCC-CCL-81), Huh7 (Charles M. Rice, The Rockefeller University), and mouse embryonic fibroblasts (MEFs, derived based on^53^) were maintained in DMEM supplemented with 10% fetal bovine serum (FBS), 100 U/mL penicillin, 0.05 mg/mL streptomycin, 0.29 mg/mL L-glutamine, and 1 mM sodium pyruvate unless otherwise specified. All cell incubation were performed at 37 °C in 5% CO_2_ unless otherwise specified.

### Virus stocks

Stocks of 17D-204 and Angola71 were generated from infectious clones by electroporating in vitro transcribed RNA into Vero cells as described previously.^16^ Virus-containing supernatant were harvested after incubation for 4 (Angola71) or 7 days (17D-204), followed by clarification by centrifugation at 875*g* for 30 min and stored at −80 °C. Virus stock titers were quantified by plaque assay on Huh7. Viruses were diluted to appropriate titer using virus diluent—PBS supplemented with 1% donor bovine serum, 100 U/mL penicillin, and 0.05 mg/mL streptomycin.

### Mouse experiments

AB6 and AGB6 mice were bred in-house. C57BL/6 mice were purchased from Charles River. Groups of randomized 6-week-old sex-matched mice were infected subcutaneously with 1 × 10^4^ PFU or mock at the hind-limb footpad after isoflurane anesthesia. Weight and swelling of footpad were monitored daily for at least 21 days. Animal experiments were not blinded. To harvest tissues, anesthetized mice were bled at submandibular vein and then sacrificed with isoflurane overdose, followed by cardiac perfusion with virus diluent. Blood were collected in Microtainer serum separator tube (BD, Cat.: 365967) and serum were obtained by centrifugation at 13523*g* for 5 min at 4 °C. Tissues were stored in virus diluent or Tri Reagent-LS (Invitrogen) at −80 °C until further usage. For histology, mice were perfused with virus diluent and 4% paraformaldehyde (PFA), followed by 24 h fixation in 4% PFA at 4 °C, prior to processing for sectioning. For antibody depletion experiments, 3 doses of 75 μg of mouse IgG2a (C1.18.4) or NK1.1 (PK136, BioXCell) or 3 doses of 150 μg of CD4 (GK1.5) or CD8 (2.43) at −3, −1, and 1 dpi were injected to animals by intraperitoneal route. IgG2a, CD4, and CD8 antibodies were generated from the corresponding hybridomas (ATCC) using CELLine bioreactors (Argos Technologies) according to manufacture’s protocol and purified with ammonium sulfate precipitation as described previously.^54^

### Flow cytometry

Popliteal (draining) lymph node were harvested from infected animals at 3 dpi, minced, and strained through 70 μm cell strainer (Falcon). Single cell suspensions were cultured in media – RPMI 1640 supplemented with 10% FBS, 100 U/mL penicillin, 0.05 mg/mL streptomycin, 20 μM β-mercaptoethanol (Sigma), and 5 μg/mL Brefeldin A (Tonbo bioscience) for 6 h. Cells were stained as previously described.^16^ Antibody and dyes used in this study were: Fixable Viability dye UV Blue (eBioscience), anti-mouse CD16/32 (93, eBioscience), APC-Cy7-anti-CD8 (2.43, Tonbo bioscience), PerCP-Cy5.5-anti-CD4 (GK1.5, Tonbo bioscience), APC-anti-NK1.1 (PK136, Tonbo bioscience), PE-anti-γδTCR (GL3, eBioscience), and FITC-anti-IFN-γ (XMG1.2, Tonbo bioscience). FITC-rat IgG1 (MOPC-21, Tonbo bioscience) is used as isotype control. Stained cells were fixed in 1% PFA and analyzed using BD LSRFortessa (BD Bioscience) and FlowJo software (Tree Star).

### Plaque assay

Huh7 cells were infected with serially diluted inoculum for 1 h. For quantification of virus in mouse organs, tissues were homogenized in sterile pestles (Bel-Art), and the virus-containing supernatant were clarified by centrifugation at 13523*g* for 15 min at 4 °C and used as inoculum. Infected Huh7 were overlaid with media supplemented with 0.5% carboxymethocellulose (CMC, high viscosity, Sigma). At 4 dpi (virus stocks) or 5 dpi (tissues), plaques were visualized by staining with 0.5% crystal violet in 2% PFA.

### Cytokine and gene expression analyses

Serum cytokines were quantified by the Cytokine 20-plex Mouse Panel (Invitrogen) according to manufacturer protocol. To analyze gene expression in different organs, RNA from tissues was isolated using Tri reagent-LS (Invitrogen) following the manufacturer protocol. Polyacryl carrier was added to serum and lymph node samples only. Reverse transcription of 100 ng of purified RNA was performed using random hexamer and TaqMan reverse transcription reagent (AB) under the following condition: 25 °C for 10 min, followed by extension at 48 °C for 30 min, and inactivation of enzymes at 95 °C for 5 min. Quantitation of 18S cDNA and antiviral genes was performed in Maxima Probe qPCR Master Mix (Thermo) and Maxima SYBR Green/Rox qPCR mix, respectively. Fluorescent intensity data were collected on a 7900HT real-time PCR system (AB). Thermocycling conditions were as follow: denaturing and polymerase activation at 95 °C for 10 min, followed by 40 cycles of denaturing at 95 °C for 15 s and extension at 60 °C for 1 min with fluorescent intensity data collection. An additional melting curve cycle was added for quality controls for antiviral genes. Primers and probes used in PCR were listed in Table S1.

### Histology

For Hematoxylin & Eosin (H&E) staining, PBS- and PFA-perfused mouse organs were harvested. Sectioning and staining were performed by the Histology Core at the McGowan Institute of Regenerative Medicine at the University of Pittsburgh. For immunostaining, PBS-perfused mouse organs were harvested fresh, sunk in 30% sucrose at 4 °C, and frozen in O.C.T. medium. For Angola71-infected mice, PFA perfusion and fixation were performed prior to sucrose treatment. Frozen tissues were stored at −80 °C until cryosection. 7 μm (spleen and liver) or 20 μm (brain) sections were permeabilized in 100% methanol at −20 °C, rehydrated in PBS with 0.05% Tween20 (PBST), antigen retrieved by proteinase K digest, and blocked in 2% BSA, 1:200 anti-CD16/32, 5% normal rat serum and goat serum, followed by incubation in M.O.M. reagent (VectorLab). YFV proteins were stained using heat-inactivated immune sera from immunized AB6 mice at 21 dpi or normal serum as isotype control at 1:50 dilution, followed by AlexaFluor488- or AlexaFluor594-conjugated goat-anti-mouse antibody. For staining liver, tissues sections were treated with normal mouse serum prior to incubation in M.O.M. reagent. Specific cell types were probed with antibodies against F4/80 (BM8, Tonbo Bioscience), CD169 (MOMA-1, Acris Antibodies, Cat.: SM066B), LYVE-1 (Goat polyclonal, R&D systems, Cat.: AF2125), and GFAP (Chicken polyclonal, Abcam, Cat.: Ab4674). Stained slides were mounted on DAPI in SlowFade Gold (Invitrogen) and imaged using FluoView 1000 confocal microscope (Olympus).

### IFN-γ ELISA

ELISA for IFN-γ was performed using the ELISA Ready-Set-Go kit (eBioscience) according to manufacture directions.

### Generation of bone marrow-derived cells

Bone marrow-derived macrophages (BMMΦ) and dendritic cells (BMDC) were generated as described previously.^19^ After 6 days of maturation, DCs were collected from supernatant and macrophages were scraped from plates. Cells were washed, counted, and seeded on U-bottom 96-well plates for DC or 24-well plates for macrophages, followed by treatment with IFNs and virus infection.

### Virus infection

Mouse embryonic fibroblasts (MEFs), macrophages, and dendritic cells were treated with various concentrations of IFN-γ (Peprotech) or IFN-α_4_/β (made in-house as described previously^55^) for 12 h. Media containing IFNs were removed prior to infection with YFVs at designated MOI for 1 h. Cells were washed three times after infection. For BMDC, cells were pelleted at 219*g* for 5 min at 4 °C between washes. Infected cells were maintained in their corresponding media. Supernatant was harvested daily for focus-forming assay.

### Focus-forming assay

1 × 10^4^ Vero cells were seeded on 96-well plate, followed by infection with serially diluted virus inoculum. After 1 h infection, cells were washed and overlaid with media supplemented with 0.5% CMC for 96 h. Cell monolayers were washed in PBS and fixed in 4% PFA for 24 h. Fixed cells were washed and permeabilized with 100% methanol at −20 °C for 10 min, followed by washes in PBS containing 0.05% tween20 (PBST). Nonspecific protein binding was blocked by incubation in PBST with 1% BSA and 5% normal goat serum for 1 h at room temperature. YFV proteins were stained using heat-inactivated immune sera from immunized AB6 mice at 21 dpi at 1:100 dilution for 24 h at 4 °C, followed by probing with HRP-goat anti-mouse antibody for 1 h at room temperature. Foci were visualized by 3,3’-diaminobenzidine as substrate (ThermoFisher, cat.: 34065).

### Statistical analysis

All data were analyzed with GraphPad Prism software. Log-rank test was performed on survival studies. Multiple *t*-test with Holm–Sidak correction was performed whenever appropriate. No statistical methods were used to ensure adequate power. Sample sizes were chosen based on experience on morbidity and mortality of YFV in mice to achieve statistical significance yet utilize fewest animals.

### Data availability statement

All relevant data from this study are available from the authors.

## Electronic supplementary material

Supplementary Information
