# Supplementary material for: Gamma-interferon exerts a critical early restriction on replication and dissemination of yellow fever virus vaccine strain 17D-204
Source: NPJ Vaccines. 2018 Jan 23;3:5. doi: 10.1038/s41541-017-0039-z (PMC5780476; doi:10.1038/s41541-017-0039-z)
Supplement: Supplementary file 1 — Supplementary Information [file 41541_2017_39_MOESM1_ESM.pdf]

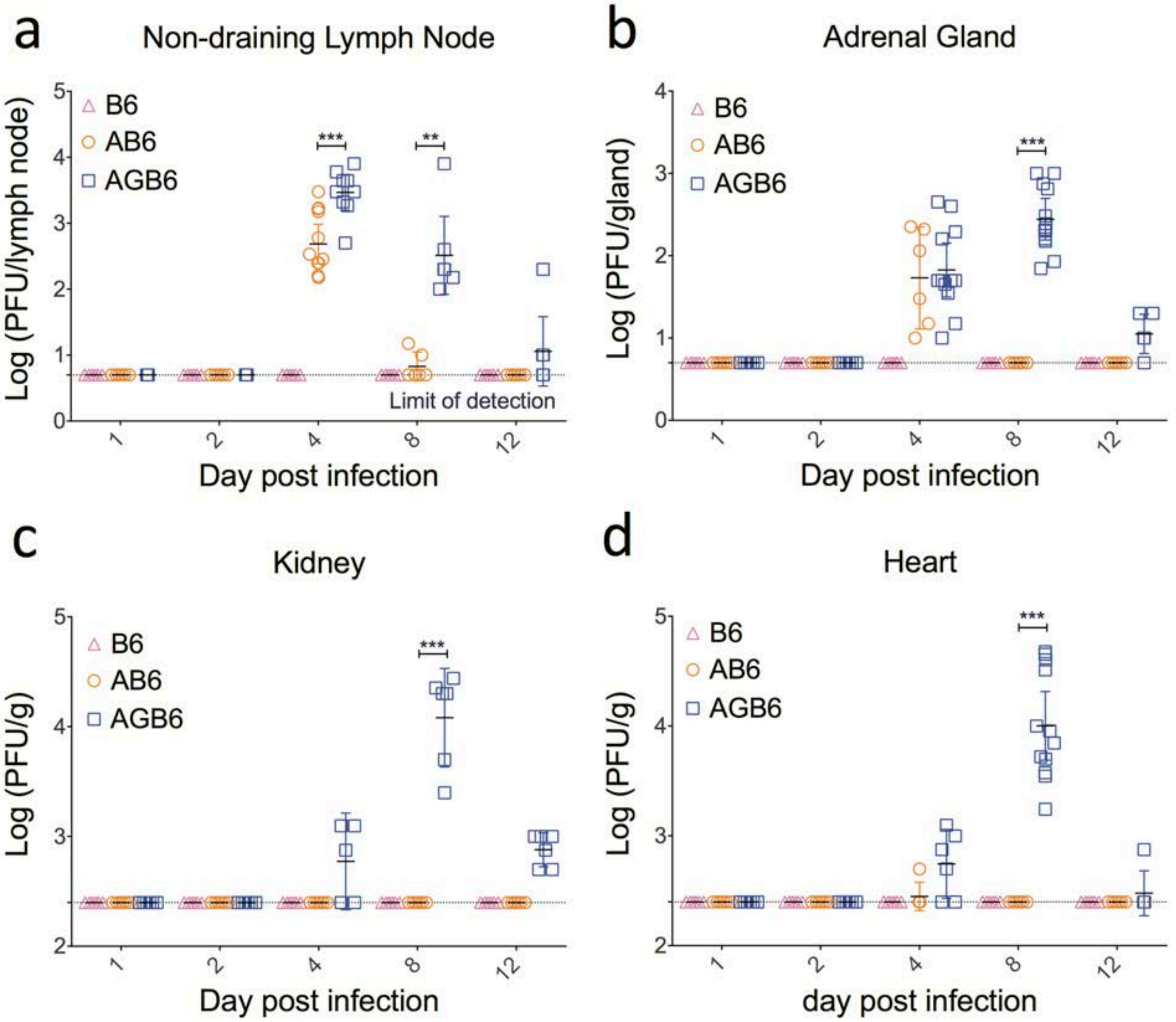

Supplemental figure 1. *In vivo* growth kinetics for 17D-204 in AB6 and AGB6 mice**.** Various tissues from 17D-204-infected mice were harvested at various time post-infection. Tissues were homogenized, and virus titers were quantified with standard plaque assay on Huh7. (a) Non-draining (mesenteric) lymph node, (b) adrenal gland, (c) kidney, (d) heart. Data are presented in geometric mean ± 95% CI. n=6, 1 out of 3 independent experiment is shown. (*: $p < 0.05$; ** $p < 0.01$; *** $p < 0.005$; **** $p < 0.001$; multiple t-test, corrected by Holm-Sidak method.)

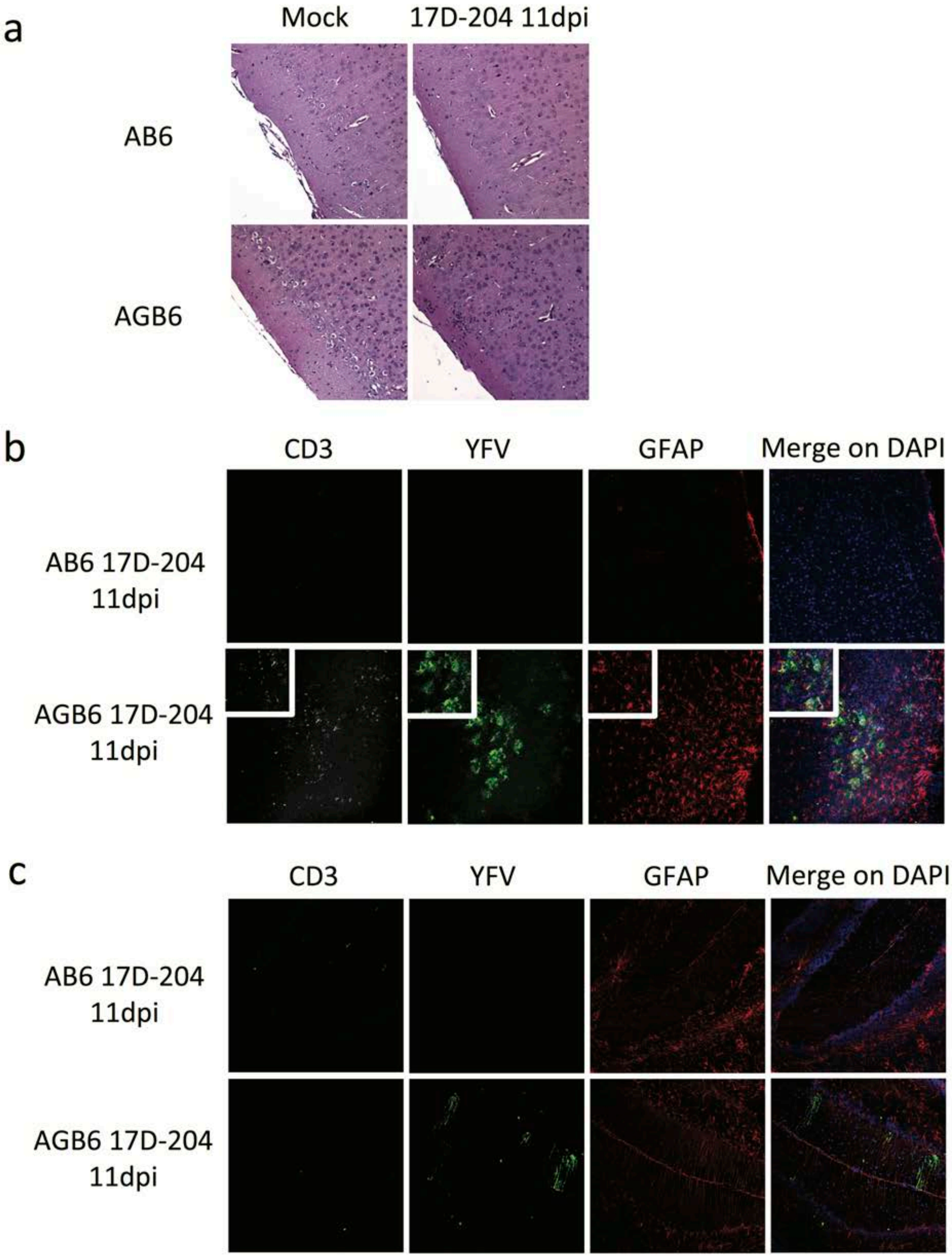
a
Mock
17D-204 11dpi
AB6
AGB6
b
CD3
YFV
GFAP
Merge on DAPI
AB6 17D-204 11dpi
AGB6 17D-204 11dpi
c
CD3
YFV
GFAP
Merge on DAPI
AB6 17D-204 11dpi
AGB6 17D-204 11dpi

Supplemental figure 2. Inflammation and viral antigens in brains of 17D-204-infected AGB6 mice. In H&E stained brain sections (a), 17D-204-infected AGB6 mice but not AB6 or mock-infected animals had increase cellular infiltration to the cerebral cortex at 11dpi. Concomitantly, YFV antigens can be detected in (b) cerebrum and (c) cerebellum. n=4, original magnification: (a-c) 20x, inset in (b) 40x.

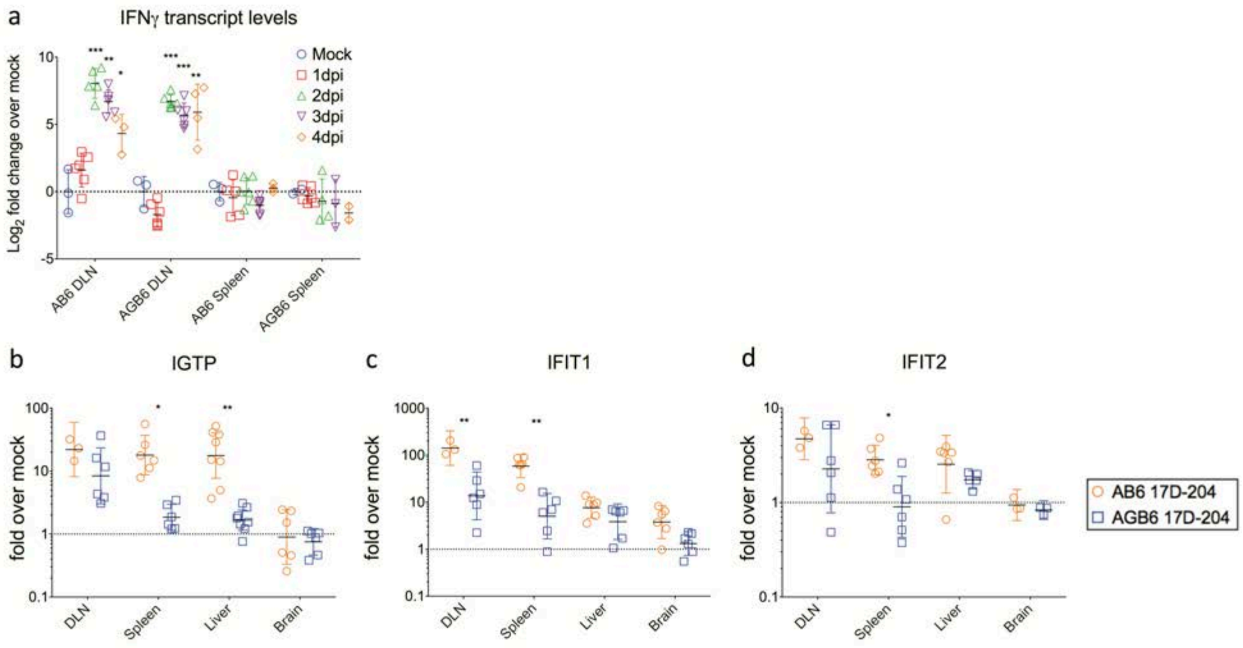

Supplemental figure 3. IFN-γ is produced early locally at draining lymph node but its downstream antiviral effect is widespread. (a) Kinetics of IFN-γ induction from 1-4dpi. (b-d) Expression of antiviral genes on 4dpi. Data are presented in geometric mean ± 95% CI. (*: p<0.05, ** p <0.01, *** p<0.005, **** p<0.001). (a) n ≥ 5. (b-d) n = 3-6.

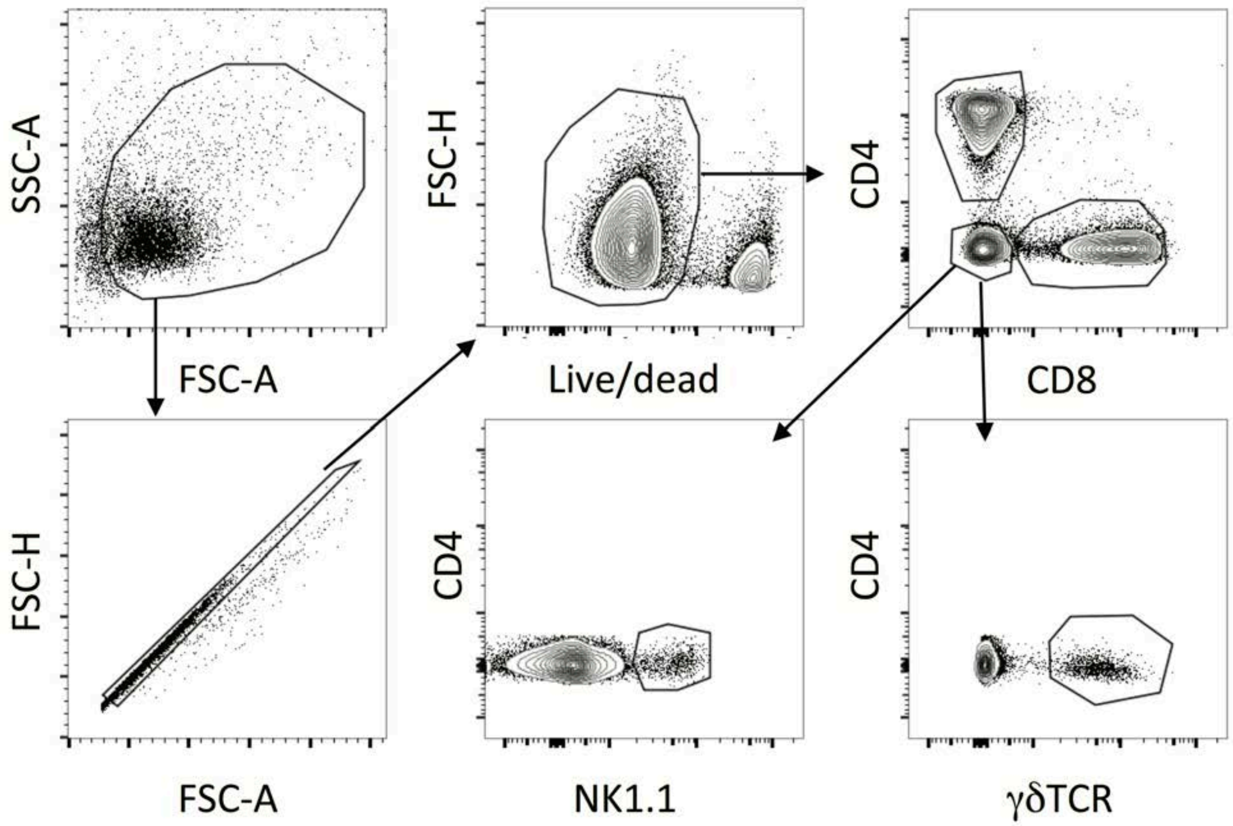

Supplemental figure 4. Gating strategy for flow cytometry analysis. Draining lymph nodes of 17D-204-infected mice were harvested at 3dpi. Single cell suspensions were prepared for flow cytometry analysis to identify cell types producing IFN-γ. Singlet live cells were gated on CD4 and CD8. Single positive cells were identified as $CD4^+$ and $CD8^+$ T cells respectively. Double negative cells were further gated on NK1.1 and γδTCR to identify NK cells and γδ T cells.

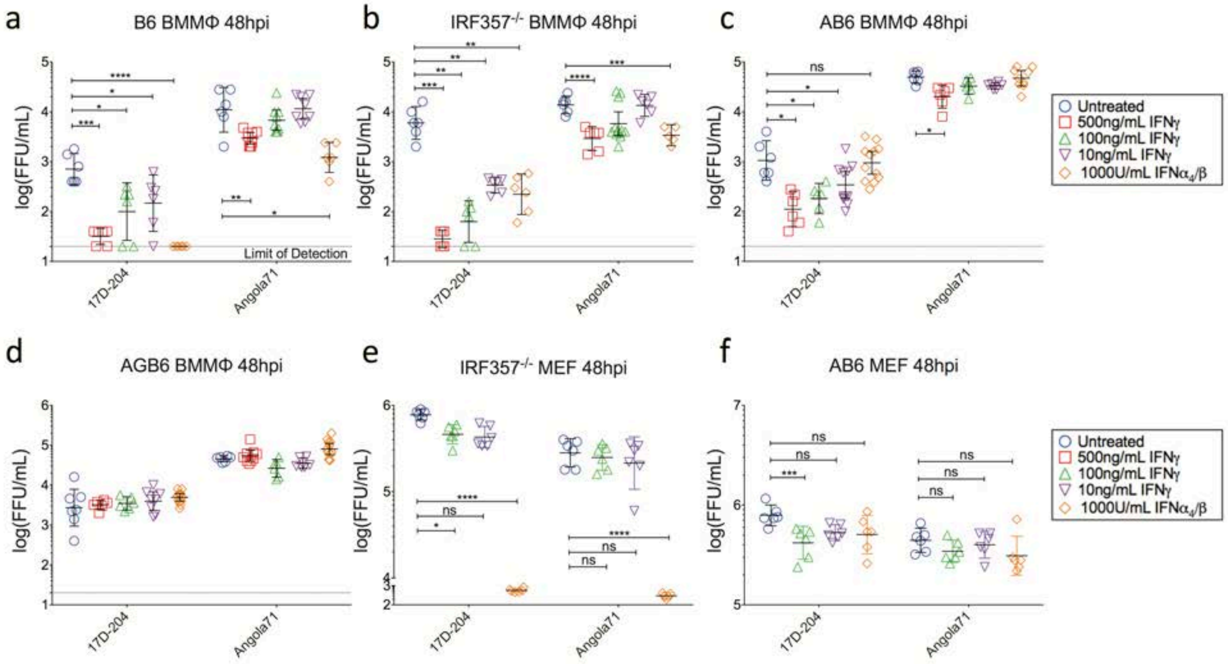

Supplemental figure 5. Robust dose-dependent inhibition of YFV by IFN-γ in myeloid cells. Indicated cell types were treated with 1000IU/mL IFN-α_4/β or various concentrations of IFN-γ 12hrs prior to infection with 17D-204 or Angola71 at MOI=0.1. At 48hpi, supernatants were harvested and infectious virus particles were quantified by focus forming assay on Vero cells. (a-d) Bone marrow-derived macrophages and (e-f) mouse embryonic fibroblasts from various strains of mice. Foci titer data were presented in geometric mean ± 95% CI. Corresponding normalized data are presented in Fig. 5. (*: p<0.05, ** p <0.01, *** p<0.005, **** p<0.001; multiple t-test, corrected by Holm-Sidak method.)

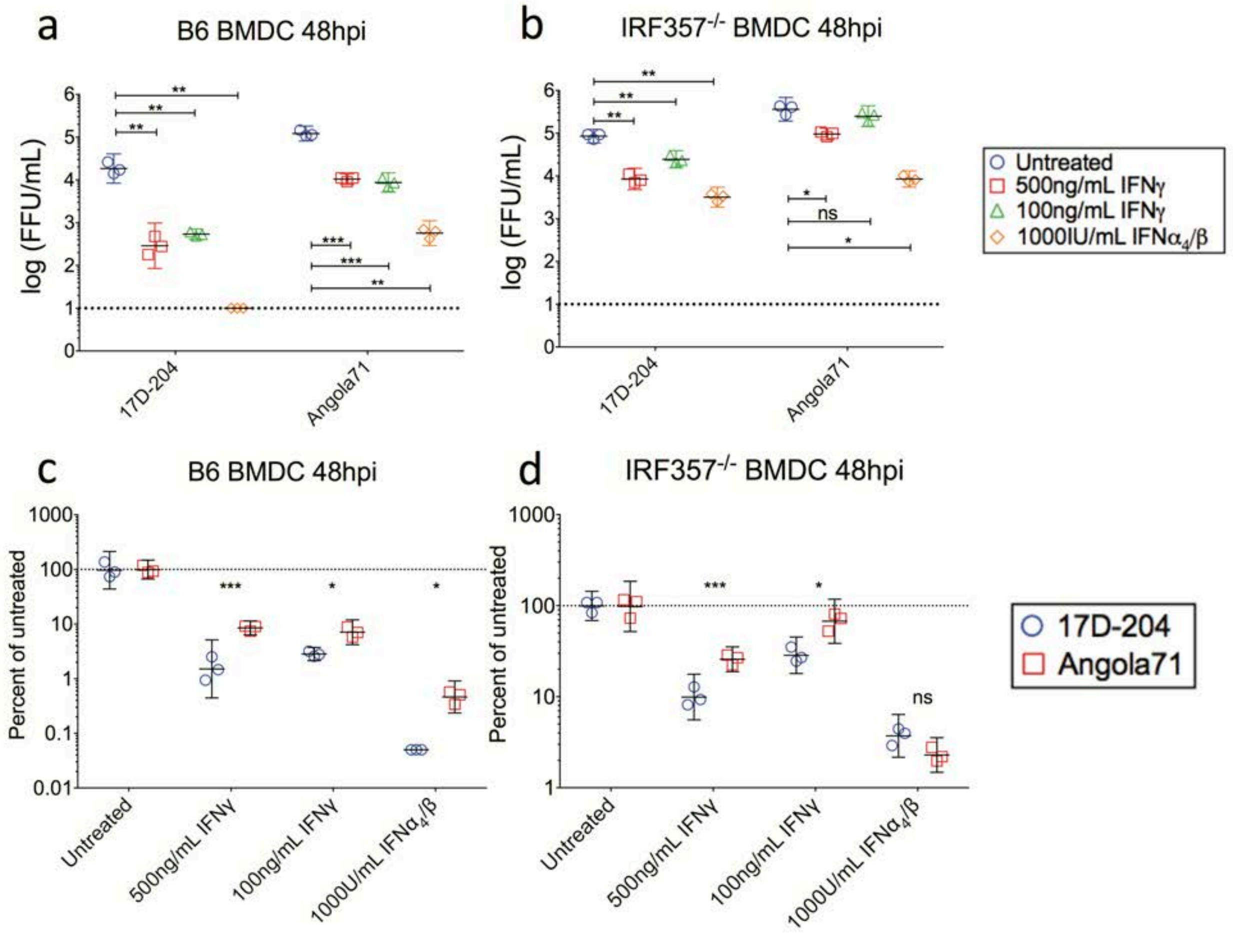

Supplemental figure 6. In dendritic cells, 17D-204 is more sensitive to IFN-γ-induced antiviral states than wild-type strain Angola71. Indicated cell types were treated with 1000IU/mL IFN-α_4/β or various concentrations of IFN-γ 12hrs prior to infection with 17D-204 or Angola71 at MOI=1. At 48hpi, supernatants were harvested and infectious virus particles were quantified by focus forming assay on Vero cells. (a-b) Foci data were presented in geometric mean ± 95% CI (c-d) Foci data from corresponding experiments were normalized to untreated cells and presented in percentage, mean ± SD. (*: $p<0.05$, ** $p<0.01$, *** $p<0.005$, **** $p<0.001$; multiple t-test, corrected by Holm-Sidak method.)

Supplemental table 1: Primer and probe sequences used in this study.

Primer/probes	Sequence
18S-S	CGCCGCTAGAGGTGAAATTCT
18S-AS	CGAACCTCCGACTTTCGTTCT
18S probe (HEX)	/5HEX/CAA GAC GGA /ZEN/CCA GAG CGA AAG CAT TTG /3IABkFQ/
mIFIT1-S	GTGGCTCACATAGAGCAGGA
mIFIT1-AS	AGTTTCCTCCAAGCAAAGGA
mIFIT2-S	AGAATTCACCTCTGGATGGG
mIFIT2-AS	GTCAAGCTTCAGTGCCAAGA
mIGTP-S	TCTGAGCAGGTTCTGAAGGA
mIGTP-AS	TCCTCGGCTTCTTTCTTCTC
mIFNγ-S	CAAAAGGATGGTGACATGAA
mIFNγ-AS	TTGGCAATACTCATGAATGC
